# Modified minimally invasive laparoscopic peritoneal dialysis catheter insertion with internal fixation

**DOI:** 10.1080/0886022X.2022.2162416

**Published:** 2023-01-12

**Authors:** Xingzhe Gao, Zhiguo Peng, Engang Li, Jun Tian

**Affiliations:** Department of organ transplantation, Qilu Hospital of Shandong University, Jinan, PR China

**Keywords:** Peritoneal dialysis, peritoneal dialysis catheter placement, laparoscopy, fixation

## Abstract

**Background:**

Laparoscopic technique is widely used in peritoneal dialysis (PD) catheter placement. We developed a modified minimally invasive laparoscopic PD catheter (PDC) insertion with internal fixation and evaluated the early results by observing the intraoperative and postoperative conditions of the novel technique with those of conventional open surgery.

**Methods:**

Retrospective research was performed on 59 patients who underwent PDC insertion from June 2019 to January 2022, including 23 patients who received open surgery and 36 patients who received modified minimally invasive laparoscopic surgery. Information such as preoperative conditions, operation time, incision length, incidence of intraoperative complications, time from operation to starting PD, time from operation to discharge, and incidence of catheter-related complications were collected and analyzed.

**Results:**

The incision length, intraoperative blood loss, catheter migration rates and the total incidence of complications 6 months after operation in the laparoscopic group were lower than those in the conventional group. There were no statistically significant differences between the two groups in operation time, time from operation to starting PD, time from operation to discharge and the incidence of catheter blockage, leakage, exit-site infection, peritoneal dialysis associated peritonitis and hernia.

**Conclusions:**

Modified minimally invasive laparoscopic PDC insertion and internal fixation method achieved direct vision and reliable fixation of the catheter, significantly reduced incision length and blood loss. The incidence of catheter migration was significantly lower than that of open surgery. Our primary findings reveal that modified minimally invasive laparoscopic PDC insertion with internal fixation is safe, effective and beneficial for PD patients.

## Introduction

Peritoneal dialysis (PD) is a kidney replacement therapy (KRT) for patients with end stage kidney disease (ESKD). PD affords greater patient autonomy and quality of life, and is more cost-effective than hemodialysis (HD) as the initiating KRT therapy with lower costs and less burden on patients and society’s medical resources, making it more suitable as the first choice of KRT [1]. Patients who are living far away from HD centers are more likely to choose PD [2].

It is of great significance to provide a quality peritoneal access with the insertion of PD catheter with minimum complications for PD patients. The economic advantages are lost if a patient switches to HD during the first year due to catheter dysfunction or serious complications [3]. Catheter insertion is thought to be the key operation to establish dialysis access for PD patients. The commonly used PD catheter (PDC) insertion methods include open surgical technique, peritoneoscopic procedure, percutaneous insertion and laparoscopic insertion [4]. Which insertion technique is actually performed should be selected according to the individual characteristics of patients, medical resources and the experience of the operators. Laparoscopic insertion technique significantly prolonged the catheter survival and decreased the risk of migration and obstruction, is becoming a widely accepted method for gaining peritoneal access [5]. The laparoscopic surgery technique for PD access is evolving rapidly, with almost as many versions of the surgery as there are surgeons performing them [6]. Most of the cases reported in the past use 10 mm laparoscopic instruments and three ports. There are many problems with this method, such as the large difference between the diameter of the port and that of the PDC, and the complex catheter fixation method [7,8]. To minimize port incision, simplify catheter fixation while maintaining good catheter function, we developed a modified minimally invasive laparoscopic PDC insertion with internal fixation. We observed and compared the postoperative complications of the new technique with those of conventional open surgery.

## Materials and methods

### Study population

Patients who received PDC placement in Qilu Hospital of Shandong University from June 2019 to January 2022 were enrolled in the retrospective study, including 23 patients undergoing conventional open surgery as the conventional group and 36 patients undergoing modified minimally invasive laparoscopic PDC insertion with internal fixation as the laparoscopic group. Standard double cuff Tenckhoff straight catheters were inserted into all patients. The laparoscopic group included 23 males and 13 females, aged 13 to 83 years, with an average of 46.92 ± 15.06 years. The conventional group included 16 males and 7 females, aged from 9 to 76 years, with an average of 45.65 ± 18.97 years.

All patients were informed in detail about the characteristics of the two surgical methods before PDC insertion and signed informed consents. Patients who were not suitable for general anesthesia performed open surgery and patients with previous abdominal surgery were recommended to perform laparoscopic surgery. All patients chose PD rather than HD for initiating KRT therapy after the latest diagnosis of ESKD and they did not receive dialysis treatment before PDC insertion. Preoperative general data were collected, including gender, age, body mass index (BMI), serum creatinine, urea nitrogen, hematological diseases and previous abdominal surgeries.

The methodology for this study was approved by the Medical Ethics Committee of Qilu Hospital of Shandong University (No.2021(265)). Informed consent was waived because of the nature of the retrospective study.

### Preoperative preparations

Polyethylene glycol electrolytes solution were used for bowel preparation preoperatively. Patients were asked to empty the bladder before the procedure, otherwise Foley catheters were inserted. A single prophylactic dose of intravenous cefazolin was given prior to the procedure.

### Laparoscopic insertion

To reserve the right iliac fossa for possible future kidney transplantation, all catheters were placed on the left side of the abdomen. Ports and catheter positions were marked on the abdominal wall before surgery. After general anesthesia, the patients were placed in supine position. Surgeries were performed by the same surgical team.

**Figure 1.**
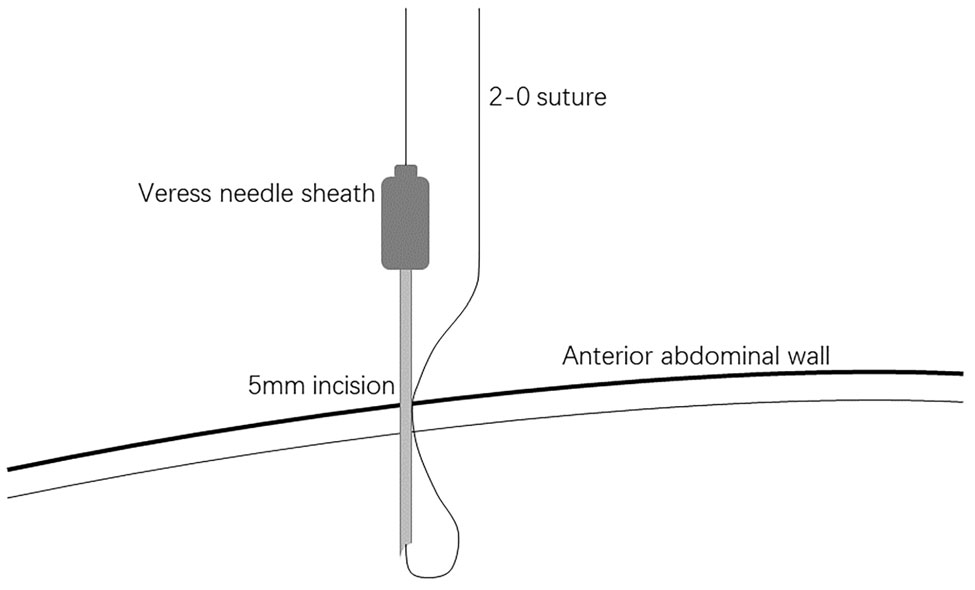
Thread the suture through the Veress needle sheath.

**Figure 2.**
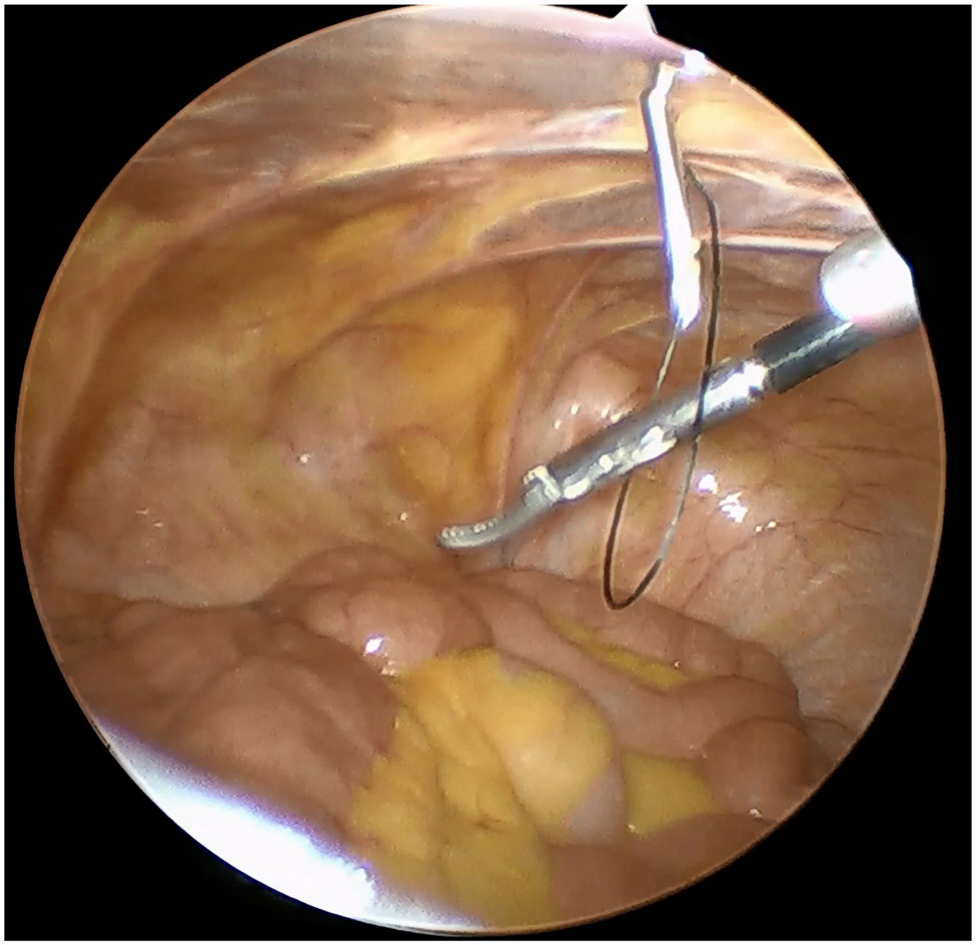
Veress needle sheath was inserted through the 5 mm incision. A forceps was used to assist the suture spread to form a ring.

**Figure 3.**
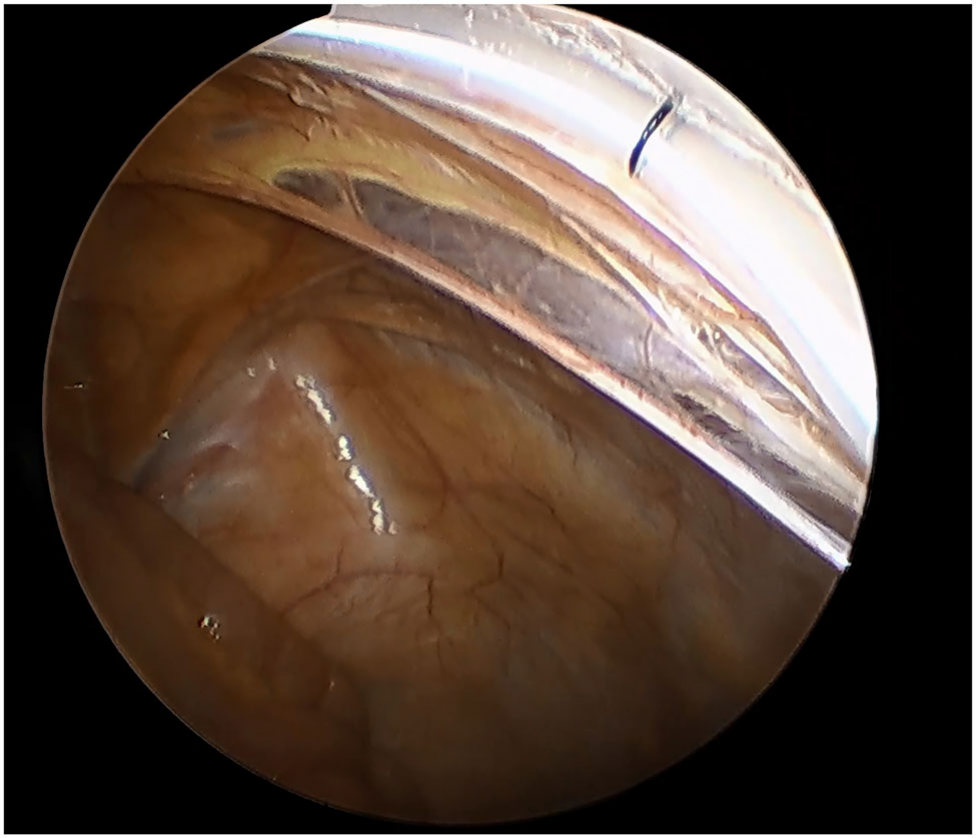
The suture was tightened and the catheter was fixed on the anterior abdominal wall.

**Figure 4.**
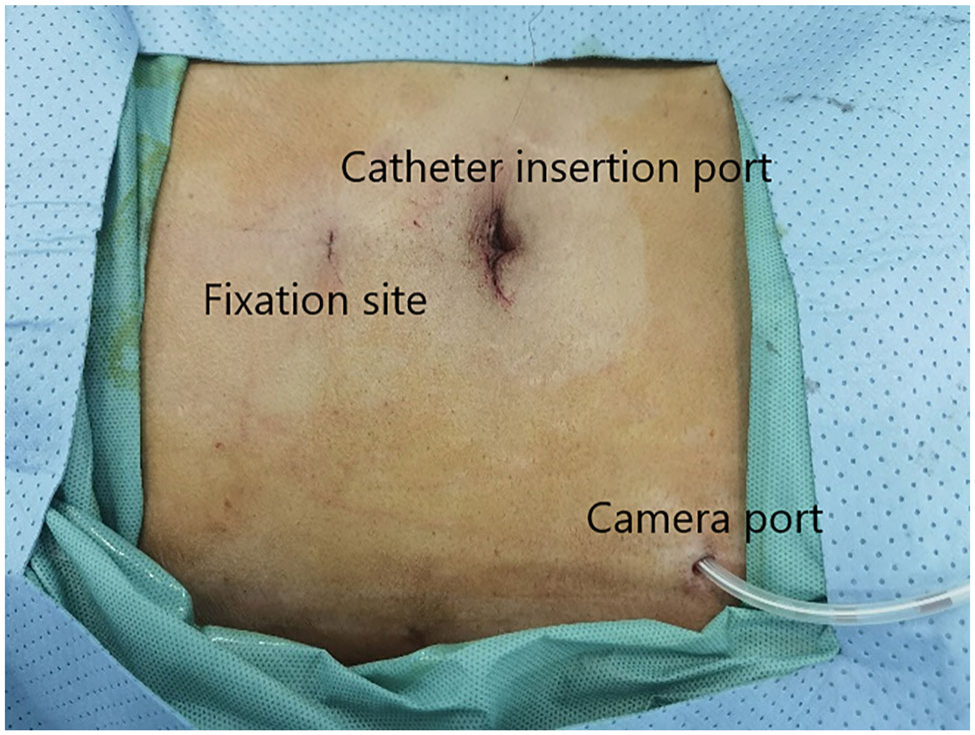
Port placements.

A small incision was made below the umbilicus. The Veress needle was inserted at this incision to establish carbon dioxide pneumoperitoneum, and the pressure was maintained at 12 mmHg. A 5 mm trocar was inserted at the initial Veress needle position. Diagnostic laparoscopy was used to examine the length of the omentum and intraperitoneal adhesions that might affect the operation. If there were no obvious abnormalities in the abdominal cavity, a second 5 mm trocar was inserted at the level of 2 cm above the umbilicus at the left margin of the rectus muscle under laparoscopic visualization. Then the second trocar was used as the laparoscopic observation port. A 2-0 suture was threaded through the Veress needle sheath, leaving half of the suture in the sheath (Figure 1). A 5 mm incision was made 5 cm below the midline umbilicus. Then the Veress needle sheath was inserted into the abdominal cavity through this 5 mm incision. Atraumatic forceps were inserted into the umbilical port to assist the suture spread to create a loop (Figure 2). Then the forceps and umbilical trocar were removed. A PDC containing a guide wire was inserted into the umbilical incision. The guide wire was shorter than 2 cm of the PDC and was used to control the catheter thread through the loop. After the catheter tail was placed in the pouch of Douglas, the inner cuff was positioned superficial to the peritoneal surface and did not enter the abdominal cavity. Then the guide wire and Veress needle sheath were removed. The suture was tightened around the catheter and fixed it on the anterior abdominal wall (Figure 3). Finally, the camera was removed and the pneumoperitoneum was released. 500 mL normal saline was used to test the patency of the catheter. A subcutaneous tunnel was established between the umbilical port and the left rectus muscle port. The distal cuff was placed between the two ports, 2–3cm from the exit site (Figure 4). Finally, all incisions were sutured before the operation was completed.

### Conventional open surgery

Local anesthesia was performed with 5 mL 2% lidocaine. The left paramidline incision was made through the skin, subcutaneous tissues, and anterior rectus sheath. The rectus muscle fibers were separated by blunt dissection to expose the posterior rectus sheath. An incision was made through the posterior sheath and peritoneum by sharp dissection, then a purse-string suture was placed around the opening. The catheter was inserted blindly into the peritoneal cavity with the help of a metal guidewire, and the catheter tip was pointed toward the bladder. After satisfactory placement has been achieved, the guidewire was withdrawn and the purse-string suture was tied. 500 mL normal saline was used to test the patency of the catheter. The inner cuff was placed within the rectus muscle and below the anterior rectus muscle sheath. A subcutaneous tunnel was established on the left abdomen. The outer cuff rested on the site 2 cm from the skin opening.

### Postoperative care

Plain film was taken 1 day after the operation to observe the catheter position. Patients initiated urgent start PD (USPD) because of obvious uremic symptoms, electrolyte disorders or fluid overload at their admission. All patients started automated PD in the supine position with 0.8 L dwell volume, 5 exchanges for 10 h per night and the volume was gradually increased up to 1.6 L by the end of 2 weeks if no complication occurred. After the patient reached full dwell volumes (1.6 L), the dialysis adequacy was assessed and PD prescription was adjusted. All patients received quality education about PD and performed at least one successful PD treatment before discharge.

### Observation index

All patients were followed up for at least 6 months. The intraoperative and postoperative conditions of the two groups were observed: operative time, incision length, intraoperative blood loss, incidence of intraoperative complications such as bleeding and visceral injury, time from operation to starting PD, and time from operation to discharge. The incidence of catheter-related complications within 6 months after operation was compared between the two groups, including catheter obstruction, catheter migration, leakage, exit-site infection, peritoneal dialysis associated peritonitis (PD peritonitis), hernia, etc. Operative time referred to the period from anesthesia completion to the last incision was sutured. Intraoperative blood loss was calculated by combining the blood volume collected within the suction canister and weighing surgical gauzes used for blood collection.

### Statistical analysis

The measurement data were expressed as Mean ± SD or median and interquartile range. T test was used for two normally distributed samples, and rank-sum test was used for non-normally distributed samples. The count data were expressed as case number (n) and percentage (%). Chi-square test and Fisher’s exact probability test were used for count data. *p* < 0.05 was considered statistically significant. All statistical analyses were conducted using SPSS 25.0 software.

## Results

### General information

There were no significant differences in gender, age, preoperative serum creatinine, preoperative blood urea nitrogen and previous abdominal surgeries between the laparoscopic group and the conventional group. The BMI in the laparoscopic group were higher than those in the conventional group, and the differences were statistically significant (*p* < 0.05) (Table 1). No patients had hematological diseases. 8 patients in the laparoscopic group had previous abdominal surgeries, including 1 cesarean section, 2 hysterectomies, 1 contraceptive operation, 1 appendicectomy, 1 kidney transplantation, 1 hernia repair and 1 ureter operation. 4 patients in the conventional group had previous abdominal surgeries, including 1 appendicectomy, 2 cesarean sections and 1 kidney transplantation.

**Table 1.** Comparison of preoperative clinical data between the two groups.

	Laparoscopic group	Conventional group	Statistics value	*p* Value
Number of cases	36	23	–	–
Gender（Male/Female）	23/13	16/7	χ² = 0.20	0.65
Age（Years）	46.92 ± 15.06	45.65 ± 18.97	t = 0.28	0.78
BMI	25.16 ± 3.51	22.73 ± 3.68	t = 2.55	0.01
Serum creatinine（umol/L）	877.08 ± 346.67	784.83 ± 337.72	t = 1.01	0.32
Blood urea nitrogen（mmol/L）	33.95 (18.91)	30.67 (10.76)	Z = -1.40	0.16
Previous abdominal surgeries	8 (22.2%)	4 (17.4%)	χ² = 0.01^a^	0.91
Previous hematological diseases	0	0	–	–

^a^indicates the corrected chi-square value.

Values presented as mean ± standard deviation, number (%), median and interquartile range.

### Comparison of intraoperative conditions

The incision length and intraoperative blood loss in the laparoscopic group were lower than those in the conventional group, and the differences were statistically significant (*p* < 0.01). There was no statistical difference in the operative time between the two groups. No intraoperative complications such as severe bleeding and visceral injury occurred in the two groups. (Table 2)

**Table 2.** Comparison of intraoperative conditions between the two groups.

	Laparoscopic group	Conventional group	Statistics value	*p* Value
Operative time（min）	55 (24)	55 (25)	Z=-1.07	0.29
Incision length（cm）	2 (0.5)	4 (0.5)	Z=-6.68	<0.01
Intraoperative blood loss（ml）	2 (1)	5 (2)	Z=-5.84	<0.01
Intraoperative complications	0 (0%)	0 (0%)	–	–

Values presented as number (%), median and interquartile range.

### Comparison of postoperative conditions

The follow-up period of the conventional group was significantly longer than that of the laparoscopic group, mainly because the novel laparoscopic technique was performed for a shorter time. The total incidence of complications within 6 months after operation in the laparoscopic group was lower than that of the conventional group (*p* < 0.05). No patients in the laparoscopic group had catheter migration, while 4 patients in the conventional group had catheter migration confirmed by imaging, and there was a statistical difference between the two groups (*p* < 0.05). No hernia occurred in the laparoscopic group, 2 cases occurred in the conventional group, but the difference was not statistical significantly. There were no significant differences between the two groups in time from operation to starting PD and postoperative to discharge. There were no statistically significant differences in the incidence of catheter blockage, leakage, exit-site infection and peritonitis between the two groups. (Table 3)

**Table 3.** Comparison of postoperative conditions between the two groups.

	Laparoscopic group	Conventional group	Statistics value	*p* Value
Postoperative follow-up (Months)	15.5 (11)	25 (20)	Z= -2.41	0.02
Time from operation to starting PD (Days)	4.5 (5)	3 (2)	Z= -1.70	0.09
Time from operation to discharge (Days)	7.5 (5)	6 (7)	Z= -0.93	0.35
Catheter obstruction	2(5.6%)	5 (21.7%)	χ²=2.14^a^	0.14
Catheter migration	0(0%)	4 (17.4%)	^b^	0.02
Leakage	2(5.6%)	0 (0%)	^b^	0.52
Exit-site infection	2(5.6%)	4 (17.4%)	χ²=1.05^a^	0.31
Peritonitis	3(8.3%)	4 (17.4%)	χ²=0.41^a^	0.52
Hernia	0(0%)	2 (8.7%)	^b^	0.15
Total complications	6(16.7%)	10 (43.5%)	χ²=5.104	0.02

^a^indicates the corrected chi-square value.

^b^indicates the Fisher’s exact probability test.

Values presented as number (%), median and interquartile range.

## Discussion

To the best of our knowledge, it is the first research to demonstrate that using Veress needle sheath and novel port placement method can reduce surgical trauma and the incidence of catheter displacement simultaneously. The follow-up results showed the safe and effective characteristic of our modified minimally invasive laparoscopic PDC insertion with internal fixation surgery. Our innovative technique needs the cooperation of nephrologists and surgeons and is worthy to be prompted in a medical center which has the ability to perform laparoscopic surgeries.

With the development of minimally invasive and laparoscopic surgery, laparoscopic PDC insertion is becoming widely accepted with minor complications and better outcomes [9]. Laparoscopic PDC insertion techniques can be divided into basic laparoscopic techniques and advanced laparoscopic techniques. Basic laparoscopic techniques are only used to observe the catheter position, while advanced laparoscopic techniques such as rectus sheath tunneling, catheter fixation, and omentum fixation are used to prevent catheter migration and dysfunction [10]. Krezalek MA et al. compared outcomes of different catheter insertion procedures. The incidence of catheter dysfunction was 31.8% with open surgery, 17.5% with basic laparoscopy, and only 4.4% with advanced laparoscopy [11]. For patients with cholecystitis and occult hernia, cholecystectomy and hernia repair can be performed during advanced laparoscopic PDC insertion to avoid postoperative infectious complications and hernia, which takes full advantage of laparoscopic procedure [12,13]. Different surgeons tend to set different numbers of laparoscopic ports. The one-port technique uses only one port to place the laparoscope, which is either a basic laparoscopic technique, or requires more incisions to insert the catheter or perform other operations. The advantages of the laparoscope are not fully utilized in the pursuit of reducing the number of ports. The three-port technique usually consists of one camera port and two operating ports, which facilitate various operations but are more invasive to the patient. In addition, these surgeons often choose to bypass the operation port to set the catheter exit site, adding other skin lesions to the patient and creating more weak sites where a hernia or leak might occur later [14,15]. In prior published research, there could be up to six skin incisions excluding catheter exit site in laparoscopy assisted PDC insertion [16]. Our modified minimally invasive PDC insertion with internal fixation is a two-port technique in advanced laparoscopic technology. We believe that the use of Veress needle sheath simplified the catheter fixation procedure and our improved port placement method makes full use of the ports. We used 5 mm laparoscopic ports, which diameter is close to that of the PDC. The catheter can effectively seal the incision to prevent pneumoperitoneum gas leakage and prevent fluid leakage caused by the mismatching of the port diameter and catheter diameter. In this study, we set the second port at the predetermined exit site so that both ports could be fully utilized and surgical injuries were reduced. Some surgeons also choose laparoscopic port wound as catheter skin exit site and the results were satisfactory [7,17,18]. The incision length, intraoperative blood loss, catheter migration rates and total complications in the laparoscopic group were significantly lower than those in the conventional group, even the BMI in the laparoscopic group was significantly higher. The difference in BMI may be due to the small sample size. All patients in the laparoscopic group were satisfied with surgical wounds, and no patients developed hernia within 6 months after surgery. The incidence of catheter blockage, peritonitis and exit-site infection in the laparoscopic group was lower than that in the conventional group. The difference, although not statistically significant, may be clinically relevant in the long run. In this study, laparoscopic surgery did not increase the operative time, time from operation to starting PD and time from operation to discharge, which would not affect patient management. Although guidelines recommend a break-in period of at least 2 weeks before PD starts, starting USPD within 2 weeks after catheter insertion has no major influence on catheter-related complications [19]. To minimize the risk of leakage, these patients started automated PD in the supine position with a small-volume PD prescription. All patients received quality education about PD operative skills and performed at least one successful PD treatment by themselves before discharge. This may result in longer hospitalization days.

Among the catheter dysfunction, catheter displacement can account for 70%, which is the most serious problem affecting the catheter function [20]. In order to prevent catheter migration, different catheter fixation methods can be performed such as establishing a rectus sheath tunnel, fixing the catheter to the abdominal wall and suturing the catheter to the pelvic tissue [21]. In our procedure, the Veress needle and the first trocar were inserted at the lower umbilical margin, the same position as the initial port in most laparoscopic surgeries, which helped the surgical team to acquire the operation skills more quickly. The incision scar can be hidden in the natural folds of the skin, which improves the esthetic degree. Because this is the linea alba, rectus sheath tunnel can’t be created in our surgery. Suturing the catheter tail to the pelvic tissue often requires two operating ports, and suturing increases the likelihood of organ injury and intraoperative bleeding. Sutures may corrode and damage pelvic tissue, and the catheter’s low mobility reduces the catheter’s ability to float freely into the deepest area of the pelvic cavity. When the catheter needs to be removed, laparoscopic surgery is often required to cut sutures [10]. Therefore, in order to minimize the number of laparoscopic ports, fix the catheter effectively and facilitate the removal of the catheter, we chose to use silk to suspend the catheter and fix it on the anterior abdominal wall. This method can not only fix the general orientation of the catheter, but also allow the catheter to float with the dialysis fluid slightly, and ensure adequate drainage on the premise of preventing catheter migration. The catheter can be easily removed by separating the inner and outer cuff under local anesthesia without the need to cut internal sutures. The fixed catheter was close to the anterior abdominal wall and as far away from the omentum as possible to reduce obstruction. No catheter migration occurred in patients receiving minimally invasive laparoscopic PDC insertion, which was significantly better than that in the conventional group, revealing the safety and effectiveness of our method. A variety of catheter suspending fixation methods have been reported, such as the use of needle passer, fascia closure instrument, hernia forceps and endo-close devices [7,8,17,22,23]. These techniques are complex to operate, traumatic to the patient and many of them need special instruments. Veress needle is widely used in laparoscopic surgery to establish pneumoperitoneum, which first appeared in the 1930s [24]. Veress needle is easy to use and can be inserted into the abdominal cavity safely with minimally invasion. It is the essential accessory in our procedures and in most laparoscopic surgeries. In our study, only a 5 mm incision was made in the lower abdominal wall, and Veress needle sheath carrying silk takes only one puncture to form a loop. This process did not require the assistance of laparoscopic instrument, which eliminates the need for additional ports and other special instruments. Another study also reported the effective use of Veress needle in helping reduce surgical trauma in percutaneous PDC insertion [25]. Neither intraoperative complications nor leakage occurred in any of the cases despite the early start on dialysis. Veress needle can also be used for skin puncture in replacement of the puncture needle in percutaneous PDC insertion by modified Seldinger technique [26]. Percutaneous technique is also a minimally invasive procedure that is widely used in placing PDCs. It has a characteristic of small incision that permits USPD and relatively straightforward technical requirements that can even be performed bedside. Percutaneous PDC insertion often uses Seldinger technique under ultrasound guidance. The Seldinger technique has its inherent complications such as unsuccessful puncture, bleeding and visceral injury because of blind penetration, especially for patients who have previous abdominal surgeries that may cause intraperitoneal adhesions [26]. At this point, laparoscopic PDC insertion is superior to ultrasound guided percutaneous technique because of direct observation of the abdominal cavity and PDC.

The ISPD guidelines recommended catheter fixation, adhesiolysis, omentopexy or omentectomy if necessary as standard procedure in laparoscopic PDC insertion [21]. Some physicians routinely perform omentopexy or partial omentectomy during PDC insertion. However, this procedure should be performed selectively because it may not be necessary when the omentum is short or adheres to a previous upper abdominal surgical site [6]. Adhesiolysis, omentopexy and omentectomy were not routinely performed in our operation. Laparoscopy was first used to observe the abdominal cavity. If the patient did not have severe intraperitoneal adhesion that might affect the operation and the omentum was not redundant, the original procedure was performed. One of our patients had a hysterectomy history. An adhesion was found between pelvic tissue and the anterior abdominal wall on laparoscopic examination. After our observation and evaluation, the catheter could be controlled to bypass the adhesion and the operation was successfully completed. No omentopexy or omentectomy was performed in any of our patients, which may be related to the small sample size. If it is necessary to perform adhesiolysis, omentopexy or omentectomy, another port can be added on the right abdominal wall to become a three-port laparoscopic method, which can easily perform these operations. Therefore, our minimally invasive PDC insertion takes safety, convenience and minimal incision in consideration, and still has the ability to perform complex procedures, which can bring maximum benefits to patients.

There are some limitations in our study. The research was conducted only in a single center and was neither a randomized nor a prospective one. The sample size is small because of the short period of the novel technique had carried out, which needs more cases to observe postoperative complications and prove the reliability of the new technique. Further investigations could also compare percutaneous approaches to laparoscopic techniques. Prospective multicenter randomized controlled studies are needed to apply and popularize our technique and further demonstrate the superiority of this kind of operation compared with other methods.

## Conclusions

We developed a novel laparoscopic PDC insertion technique which was characterized by less catheter migration and shorter incision length, which brought patients fewer complications and better esthetics. Our clinical observation reveals that modified minimally invasive laparoscopic PDC insertion with internal fixation is safe, effective and beneficial for PD patients.

## References

[1] Liu J, Hutton DW, Gu Y, et al. Financial implications of dialysis modalities in the developing world: a chinese perspective. Perit Dial Int. 2020;40(2):193–201. 32063196 10.1177/0896860819893812

[2] Wang V, Maciejewski ML, Coffman CJ, et al. Impacts of geographic distance on peritoneal dialysis utilization: refining models of treatment selection. Health Serv Res. 2017;52(1):35–55. 27060855 10.1111/1475-6773.12489 PMC5264105

[3] Bagul A, Thiyagarajan UM, Mamode N. Laparoscopic peritoneal dialysis catheter (PDC) insertion: does it really make a difference? J Nephrol. 2014;27(2):127–134. 24536002 10.1007/s40620-013-0031-2

[4] Crabtree JH, Chow KM. Peritoneal dialysis catheter insertion. Semin Nephrol. 2017;37(1):17–29. 28153191 10.1016/j.semnephrol.2016.10.004

[5] Chen Y, Shao Y, Xu J. The survival and complication rates of laparoscopic versus open catheter placement in peritoneal dialysis patients: a Meta-Analysis. Surg Laparosc Endosc Percutan Tech. 2015;25(5):440–443. 26429052 10.1097/SLE.0000000000000188

[6] Crabtree JH. SAGES guidelines for laparoscopic peritoneal dialysis access surgery. Surg Endosc. 2014;28(11):3013–3015. 25149640 10.1007/s00464-014-3812-3

[7] Bae IE, Chung WK, Choi ST, et al. Laparoscopic internal fixation is a viable alternative option for continuous ambulatory peritoneal dialysis catheter insertion. J Korean Surg Soc. 2012;83(6):381–387. 23230557 10.4174/jkss.2012.83.6.381 PMC3514481

[8] Li JR, Chen WM, Yang CK, et al. A novel method of laparoscopy-assisted peritoneal dialysis catheter placement. Surg Laparosc Endosc Percutan Tech. 2011;21(2):106–110. 21471803 10.1097/SLE.0b013e31820af4ad

[9] Janez J. Laparoscopically assisted insertion of peritoneal dialysis catheter. J Minim Access Surg. 2019;15(1):80–83. 29319017 10.4103/jmas.JMAS_196_17 PMC6293685

[10] Haggerty S, Roth S, Walsh D, SAGES Guidelines Committee, et al. Guidelines for laparoscopic peritoneal dialysis access surgery. Surg Endosc. 2014;28(11):3016–3045. 25294537 10.1007/s00464-014-3851-9

[11] Krezalek MA, Bonamici N, Lapin B, et al. Laparoscopic peritoneal dialysis catheter insertion using rectus sheath tunnel and selective omentopexy significantly reduces catheter dysfunction and increases peritoneal dialysis longevity. Surgery. 2016;160(4):924–935. 27524427 10.1016/j.surg.2016.06.005

[12] Janež J. Synchronous laparoscopic insertion of peritoneal dialysis catheter and cholecystectomy in patients with End-Stage renal disease and Gallstones - Our experience. Perit Dial Int. 2019;39(5):489–491. 31501294 10.3747/pdi.2019.00003

[13] Kou HW, Yeh CN, Tsai CY, et al. Clinical benefits of routine examination and synchronous repair of occult inguinal hernia during laparoscopic peritoneal dialysis catheter insertion: a single-center experience. Hernia. 2021;25(5):1317–1324. 33548007 10.1007/s10029-020-02364-7 PMC8514383

[14] Ashegh H, Rezaii J, Esfandiari K, et al. One-port laparoscopic technique for placement of tenckhoff peritoneal dialysis catheters: report of seventy-nine procedures. Perit Dial Int. 2008;28(6):622–625. 18981392

[15] Mo M, Ju Y, Hu H, et al. Peritoneal dialysis catheter emplacement by advanced laparoscopy: 8-year experience from a medical center of China. Sci Rep. 2017;7(1):9097. 28831180 10.1038/s41598-017-09596-1 PMC5567303

[16] Maheshwari PN, Heda RS, Oswal AT, et al. Laparoscopy-assisted continuous ambulatory peritoneal dialysis catheter placement using amplatz dilators: a new technique with results. Urology. 2014;84(6):1521–1524. 25432849 10.1016/j.urology.2014.08.027

[17] Musbahi A, Kanakala V. A modified laparoscopic peritoneal dialysis insertion technique, the 'one scar technique’ can minimise short and long term complications: a retrospective cohort study. J Vasc Access. 2021;22(5):744–748. 32993444 10.1177/1129729820961970

[18] Ko J, Ra W, Bae T, et al. Two-port laparoscopic placement of a peritoneal dialysis catheter with abdominal wall fixation. Surg Today. 2009;39(4):356–358. 19319648 10.1007/s00595-008-3877-5

[19] Wen X, Yang L, Sun Z, et al. Feasibility of a break-in period of less than 24 hours for urgent start peritoneal dialysis: a multicenter study. Ren Fail. 2022;44(1):450–460. 35272577 10.1080/0886022X.2022.2049306 PMC8920377

[20] Yilmazlar T, Kirdak T, Bilgin S, et al. Laparoscopic findings of peritoneal dialysis catheter malfunction and management outcomes. Perit Dial Int. 2006;26(3):374–379. 16722032

[21] Crabtree JH, Shrestha BM, Chow KM, et al. Creating and maintaining optimal peritoneal dialysis access in the adult patient: 2019 update. Perit Dial Int. 2019;39(5):414–436. 31028108 10.3747/pdi.2018.00232

[22] Shen Q, Jiang X, Shen X, et al. Modified laparoscopic placement of peritoneal dialysis catheter with intra-abdominal fixation. Int Urol Nephrol. 2017;49(8):1481–1488. 28455661 10.1007/s11255-017-1593-z

[23] Rouse M, Choi J, Bakhit J, et al. Laparoscopic EndoClose fixation of a peritoneal catheter reduces migration. ANZ J Surg. 2020;90(1-2):72–75. 31786820 10.1111/ans.15506

[24] Veress J. Neues instrument zur ausführung von brust- oder bauchpunktionen und pneumothoraxbehandlung. Dtsch Med Wochenschr. 1938;64(41):1480–1481.

[25] Pethő Á, Szabó RP, Tapolyai M, et al. Bedside placement of peritoneal dialysis catheters - a single-center experience from Hungary. Ren Fail. 2019;41(1):434–438. 31162993 10.1080/0886022X.2019.1614058 PMC6566899

[26] Zou Y, Ma Y, Chao W, et al. Assessment of complications and short-term outcomes of percutaneous peritoneal dialysis catheter insertion by conventional or modified seldinger technique. Ren Fail. 2021;43(1):919–925. 34092201 10.1080/0886022X.2021.1925296 PMC8189143

